# Stress native mapping does not distinguish patients with previous myocardial infarction with non‐obstructive coronary arteries from healthy volunteers

**DOI:** 10.1111/cpf.70070

**Published:** 2026-06-05

**Authors:** Daniel F. Andersson, Rebecka Steffen Johansson, Per Tornvall, Peder Sörensson, Jannike Nickander

**Affiliations:** ^1^ Department of Clinical Physiology Karolinska Institutet and Karolinska University Hospital Stockholm Sweden; ^2^ Department of Clinical Research and Education Södersjukhuset, Karolinska Institutet Stockholm Sweden; ^3^ Department of Medicine Solna Karolinska Institutet and Karolinska University Hospital Stockholm Sweden

**Keywords:** native T1, native T2, quantitative perfusion, MINOCA, CMD, microvascular dysfunction

## Abstract

**Aims:**

Patients with myocardial infarction with non‐obstructive coronary arteries (MINOCA) may be affected by coronary microvascular dysfunction with reduced stress perfusion. Changes in native T1 and T2 reflect changes in myocardial perfusion; therefore, the aim of our study was to investigate whether non‐contrast, adenosine stress native T1 and T2 are affected in patients with previous suspected MINOCA.

**Methods and Results:**

Patients with MINOCA and a normal CMR (*n* = 15, 59 ± 7 years, 60% female) underwent 1.5T CMR together with age‐ and sex‐matched volunteers. The protocol included native T1, native T2 and quantitative perfusion mapping, at rest and during adenosine stress. Myocardial stress perfusion was globally reduced, both transmurally (2.9 ± 0.9 vs. 3.6 ± 0.7 mL/min/g, *p *= 0.02) and in the subendocardium (2.64 ± 0.81 vs. 3.47 ± 0.78 ml/min/g, *p *= 0.008) in patients with MINOCA, with a reduced global ratio of subendocardial‐to‐transmural stress perfusion (0.921 ± 0.042 vs 0.957 ± 0.039, *p *= 0.021). However, there were no differences in global transmural rest native T1 or T2, stress native T1 or T2, or ΔT1‐ or ΔT2 values between patients and volunteers. Overall, transmural myocardial perfusion correlated with native T1 (R^2^ = 0.27, *p *< 0.001) and T2 (R^2^ = 0.48, *p *< 0.001) globally, and ΔT1 correlated with the MPR globally (R^2^ = 0.15, *p *< 0.05).

**Conclusions:**

Native T1‐ and T2‐mapping during adenosine stress, although correlated with quantitative myocardial perfusion, are not alone sufficiently sensitive methods for distinguishing patients with MINOCA and reduced stress perfusion from healthy volunteers.

## 1 INTRODUCTION

Myocardial infarction with non‐obstructive coronary arteries (MINOCA) is an acute condition in which the universal definition of myocardial infarction (MI) is fulfilled in the absence of angiographically significant obstructive coronary artery disease (< 50%) (Abdu et al., 2020). Cardiac magnetic resonance imaging (CMR) has emerged as an essential, non‐invasive tool in the workup of MINOCA (Collet et al., 2021; Sörensson et al., 2021); however, many patients with MINOCA have a normal CMR scan (Sörensson et al., 2021). A normal coronary angiography and CMR scan in the setting of evident myocardial damage may be attributed to coronary microvascular dysfunction (CMD), a condition estimated to be present in up to half of patients with stable ischemia and non‐obstructive coronary artery disease (CAD) (Schumann et al., 2021). CMD encompasses a spectrum of structural and functional alterations in the coronary microcirculation which lead to a reduced myocardial vasodilatory response to increases in metabolic demand (Camici et al., 2015; Schumann et al., 2021). CMD can be detected using quantitative CMR perfusion mapping, which can assess microvascular function (Groepenhoff et al., 2021) by quantifying myocardial perfusion at rest and during adenosine stress using first‐pass perfusion imaging with gadolinium‐based contrast agents (Brown et al., 2022; Kellman et al., 2017; Kotecha et al., 2019).

Current recommendations encourage the use of non‐contrast (native) techniques at rest whenever possible (Kramer et al., 2020), and native T1 imaging can differentiate between normal, ischemic and infarcted myocardium (van Assen et al., 2019; Yimcharoen et al., 2020). Native T1 and T2 have been demonstrated to increase during adenosine stress in healthy subjects, which can be attributed to changes in myocardial perfusion (Nickander et al., 2019), and have been shown to correlate with myocardial perfusion in patients with severe Covid‐19 (Nickander et al., 2023). It has been recently shown that patients with MINOCA and normal CMR findings at index hospitalization have reduced myocardial perfusion at a follow‐up stress CMR compared to healthy volunteers (Steffen Johansson et al., 2023). However, it is currently unknown if non‐contrast CMR techniques alone can provide a diagnosis in this subgroup of patients with MINOCA. Therefore, the aim of this study was to investigate if stress native T1 and T2 mapping can differentiate patients with MINOCA and a previously normal CMR examination from healthy age‐ and sex‐matched volunteers without symptoms of ischemic heart disease (IHD).

## 2 METHODS

### 2.1 Study group

Patients with MINOCA (*n* = 15) were identified from the SMINC‐2 (Stockholm Myocardial Infarction with Normal Coronaries 2) study (*n* = 148) (Sörensson et al., 2021), in which patients with MINOCA were included between 2014 and 2018. Patients were consecutively included for follow‐up CMR in the present study if the initial CMR scan as part of SMINC‐2, performed 2–4 days following hospital admission at index event, as well as a 6‐month follow up exam (Nickander et al., 2023), showed normal findings as read by two independent level 3 observers. A normal CMR scan was defined as a structurally normal heart with normal extracellular volume (ECV), no wall‐motion abnormalities, no oedema on T1 mapping and no late gadolinium enhancement (LGE) (Sörensson et al., 2021). SMINC‐2 inclusion criteria (Sörensson et al., 2021) included fulfilment of the fourth universal definition of MI (Thygesen et al., 2018) in patients with non‐obstructive (stenosis ≤ 50% of coronary vessel diameter) coronary arteries as determined visually on coronary angiography. Exclusion criteria for SMINC‐2 included lack of sinus rhythm on admission electrocardiogram (ECG), pulmonary embolism, previous MI, previously known cardiomyopathy, severe chronic obstructive pulmonary disease, severe kidney impairment (S‐creatinine >150 µmol/L), pacemaker, or claustrophobia. The same inclusion and exclusion criteria applied to the present study. The time from index CMR as part of SMINC‐2 to adenosine stress CMR in the present study was a median of 5 years [3 years 164 days–6 years 309 days]. Age‐ and sex‐matched volunteers (*n* = 15) without previous history of angina pectoris, respiratory conditions, or renal disease were recruited through advertisement at the Karolinska Institutet university campus for comparison to patients with MINOCA. Study patients and volunteers were well‐matched in their clinical characteristics, asides from a more frequent use of statins in patients with MINOCA, and both patients and volunteers were free from anginal symptoms as assessed by the Seattle Angina Questionnaire at the time of follow‐up (Steffen Johansson et al., 2023) (Table 1). Using previously published data from healthy individuals (Nickander et al., 2020), an a priori power calculation determined that 16 participants would be required to detect a 0.78 ml/min/g difference in stress perfusion with 80% power and a significance level of 0.05. The study was performed in accordance with the Declaration of Helsinki and good clinical practice and was approved by the Regional Ethics Review Board in Stockholm, ID‐number 2021‐06837 and the Swedish Ethical Review Authority, ID‐ number 2014/131‐31/1, 2017/2415‐32/1, 2021‐06837‐02. All patients provided written informed consent.

**Table 1:** Clinical and CMR characteristics of patients and volunteers.

	MINOCA (*n* = 15)	Volunteers (*n* = 15)
Female sex, *n* (%)	9 (60%)	9 (60%)
Age, years	59 ± 7	59 ± 7
Height, cm	173 ± 10	173 ± 10
Weight, kg	75 ± 15	71 ± 14
BSA, m^2^	1.9 ± 0.2	1.8 ± 0.2
Creatinine, µmol/L	71 ± 17	74 ± 12
Haematocrit, %	43 ± 5	43 ± 4
Hypertension, *n* (%)	8 (57%)	3 (20%)
Diabetes mellitus, *n* (%)	2 (14%)	1 (7%)
Hyperlipidemia, *n* (%)	5 (36%)	2 (13%)
Smoking currently, *n* (%)	4 (29%)	1 (7%)
Smoking previously, *n* (%)	9 (64%)	6 (40%)
Statins, *n* (%)	7 (50%)	1 (7%)
Beta blockers, *n* (%)	3 (21%)	2 (13%)
ACE‐I/ARB, *n* (%)	6 (43%)	2 (13%)
Calcium channel blockers, *n* (%)	2 (14%)	1 (7%)
SAQ‐7 summary score	100 [97–100]^a^	100 [100–100]
R‐R, ms	996 ± 125	890 ± 103
LVM, mL	97 ± 28	89 ± 23
LVM, g	101 ± 29	93 ± 25
EDV, mL	154 ± 39	148 ± 38
ESV, mL	72 ± 26	71 ± 25
SV, mL	81 ± 18	77 ± 20
EF, %	54 ± 8	52 ± 8
CO, l/min	4.9 ± 1.0	5.2 ± 1.2
Heart rate rest (bpm)	67 ± 9	71 ± 11
SBP rest (mmHg)	134 ± 15	128 ± 15
RPP rest	9014 ± 1672	9114 ± 1781
Heart rate stress (bpm)	88 ± 10	90 ± 8
SBP stress (mmHg)	137 ± 18	128 ± 15
RPP stress	12083 ± 2265	11506 ± 1699
TnT (ng/l) at index CMR	28 [15–58]	
Maximal TnT at index event (ng/L)	82 [37–112]	
Ischemic LGE (n)	1/15	0/15

Abbreviations: ACE‐I, Angiotensin‐Converting‐Enzyme Inhibitors, ARB, Angiotensin Receptor Blockers, BSA, Body surface area, CMR, Cardiovascular Magnetic Resonance, CO, Cardiac output, EDV, End‐diastolic volume, EF, Ejection fraction, ESV, End‐systolic volume, EVF, Erythrocyte Volume Fraction, LGE, Late Gadolinium Enhancement, LVM, Left ventricle mass, RPP, Rate‐pressure product, R‐R, R‐wave to R‐wave interval, SAQ‐7, Seattle Angina Questionnaire‐7, SBP, Systolic blood pressure, SV, Stroke volume, TnT, Troponin T (presented as median [interquartile range]).

a Data missing for one patient. (Steffen Johansson et al., 2023).

### 2.2 Image acquisition

**Figure 1:**
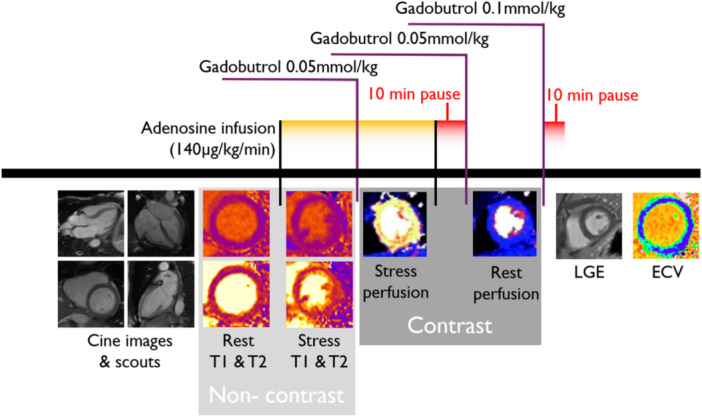
Timeline of the CMR scanning protocol. Scouts and cine images were acquired first, followed by native T1 and T2 mapping at rest. After 3 min of adenosine infusion, native T1 and T2 mapping were acquired, followed by first‐pass perfusion imaging using gadobutrol (0.05 mmol/kg), which generated a perfusion map and a blood volume map. After the adenosine infusion was terminated, a 10‐min pause followed to reach contrast equilibrium. First‐pass perfusion images were acquired using gadobutrol (0.05 mmol/kg) at rest and an additional dose of gadobutrol (0.1 mmol/kg) was administered for post‐contrast LGE and ECV maps at rest.

Patients and volunteers underwent CMR at 1.5T (MAGNETOM Aera®, Siemens Healthineers, Forchheim, Germany) with a phased‐array eighteen channel body matrix coil together with a spine matrix coil. Subjects were examined in the supine position. Blood haematocrit and creatinine were determined prior to imaging. Heart rate and blood pressure were measured at rest and during adenosine stress. An adequate stress response was defined as a heart rate increase by at least 15 beats per minute. The rate pressure product (RPP) was calculated as heart rate multiplied by systolic blood pressure (Gobel et al., 1978; White, 1999). The image acquisition protocol is shown in Figure 1.

Cine images assessing left ventricular function were acquired in one short‐axis and three long‐axis slices through retrospectively ECG‐gated balanced steady state free precession (bSSFP) with flip angle (FA) 68 degrees, pixel size 1.4 x 1.9 mm^2^, slice thickness 8.0 mm, echo time (TE)/repetition time (TR) 1.19 ms/37.05 ms, matrix size 256 x 144, field of view (FOV) 360 x 270 mm.

Quantitative myocardial perfusion maps (mL/min/g) were acquired using a first‐pass perfusion imaging research sequence as described by Kellman et al. (Kellman et al., 2017) in three short‐axis slices (basal, midventricular, apical) following an intravenous contrast agent bolus (0.05 mmol/kg Gadobutrol, Gadovist, Bayer AB, Solna, Sweden), during and after adenosine infusion (140 µg/kg/min, increased up to 210 µg/kg/min in the absence of an adequate stress response, Adenosin, Life Medical AB, Stockholm). Adenosine and gadolinium contrast were administered in separate cannulas. Typical imaging parameters for perfusion maps were FA 50 degrees, slice thickness 8.0 mm, TE/TR 1.04/2.5 ms, bandwidth 1085 Hz/pixel, FOV 360 x 270 mm and saturation delay/trigger delay (TD) 95/40 ms.

Native T1 maps were acquired in three short‐axis slices (basal, midventricular, apical) at rest, and in one midventricular short‐axis slice during adenosine stress, using the previously described method for adenosine administration. Native T1 maps were acquired in end‐diastole using an ECG‐gated Modified Look‐Locker inversion recovery (MOLLI 5s(3s)3s) single‐shot SSFP research sequence with the following parameters: flip angle 35°, pixel size 1.4 x 1.9 mm^2^, slice thickness 8.0 mm, TE/TR 1.12/2.7 ms, and a 256 x 144 matrix. Following a bolus of contrast agent (0.1 mmol/kg, Gadobutrol, Gadovist®, Bayer AB, Solna, Sweden), post‐contrast T1 maps were acquired in the same position as the rest images. Rest native and post‐contrast T1 maps were used to generate ECV maps in three short‐axis slices (basal, midventricular, apical), calibrated by haematocrit (Arheden et al., 1999; Kellman et al., 2012).

Native T2 maps were acquired in three short‐axis slices (basal, midventricular, apical) at rest and in one midventricular short‐axis slice during adenosine stress (as per the previously described method for adenosine stress) using a T2‐prepared SSFP sequence with three T2‐preparation echo times (0, 25, and 55 ms). Typical imaging parameters included: FA 70°, pixel size 1.4 x 1.4 mm^2^, slice thickness 8.0 mm, acquisition window 700 ms, TE/TR 1.06/2.49 ms, and matrix size 144 x 256. LGE images were acquired post‐contrast using a free‐breathing PSIR sequence (Kellman et al., 2005) with bSSFP readout. Typical imaging parameters included matrix size 125 x 125, voxel size 1.3 x 1.3 x 7.0 mm^3^, slice thickness 8.0 mm, FOV 340 x 276 mm, TE/TR 3.4/8.25 ms, and FA 50 degrees.

### 2.3 Image analysis

Following pseudonymisation, images were analysed offline using Segment (version 3.3 R9405e Medviso AB, Lund, Sweden) (Heiberg et al., 2010). The endo‐ and epicardial borders of the left ventricular wall were manually delineated into circumferential regions of interest (ROIs) in short‐axis image stacks (Tufvesson et al., 2015). A 10% margin of erosion for the endo‐ and epicardial borders was set to avoid partial volume effects from the blood pool and adjacent tissues. The subendocardium was separately analysed using the same delineation and 10% endocardial margin, but instead with an epicardial erosion margin of 50%. The left ventricular wall was segmented into 16 segments according to the Standardized Myocardial Segmentation of the American Heart Association (Cerqueira et al., 2002) and exported as bulls eye plots (Cain et al., 2005). Segments with outlier values (>2 SD) determined to be of poor quality due to partial volume effects or thin myocardium were excluded before averaging per‐patient values for each map (Table 4, supplementary). For equal comparison with the single‐slice stress maps, resting T1‐ and T2‐values were separately exported and analysed both as 16 segments across three slices ("Global") and as 6 mid‐slice segments only ("Mid‐slice"). Per‐patient averages of both transmural and subendocardial measurements were compared globally and between mid‐slices only. In total, 700 segments in three slices for T1‐, T2‐ and perfusion maps at rest and 262 segments in one slice for T1‐, T2‐ and perfusion during adenosine stress were included in the analysis. To assess reproducibility, segmental analysis was performed by two independent observers for all 30 study participants, of which one also repeated an analysis of a random subset of 10 patients a second time. Inter‐ and intra‐observer reliability were assessed using the intraclass correlation coefficient and was 0.92‐1.00 (*p* < 0.001) for all measurements (Table 5, supplementary).

### 2.4 Statistical analysis

Continuous variables were reported as means with standard deviations (SD). Ordinal variables were reported as numbers and percentages. The Shapiro‐Wilk test was used to assess all data for normality. In normally distributed data, a two‐tailed Students’ independent t‐test was used to compare means. Body surface area (BSA) was calculated using the Mosteller formula (Mosteller, 1987). Volumetric measurements and myocardial mass were indexed to BSA. The MPR was calculated as a ratio of peak stress perfusion to rest perfusion. ∆T1 and ∆T2 were expressed as the percentage change from rest to stress for native T1 and T2, respectively. The ratio of subendocardial‐to‐transmural stress perfusion was calculated per subject as global subendocardial stress perfusion divided by global transmural stress perfusion and compared between groups. The relationships between native T1, native T2, perfusion, ΔT1, ΔT2 and MPR were assessed globally using Pearson's linear correlation coefficient and presented as R^2^‐values. Per‐patient values and derived variables were calculated by averaging per‐subject segmental values using Microsoft Excel (Microsoft® Excel®, version 2203) and statistical analysis was performed using SPSS Statistics® (International Business Machines SPSS Statistics®, version 27.0). The significance level for all statistical analyses was set at *p* < 0.05.

## 3 RESULTS

### 3.1 Native T1 and T2

**Figure 2:**
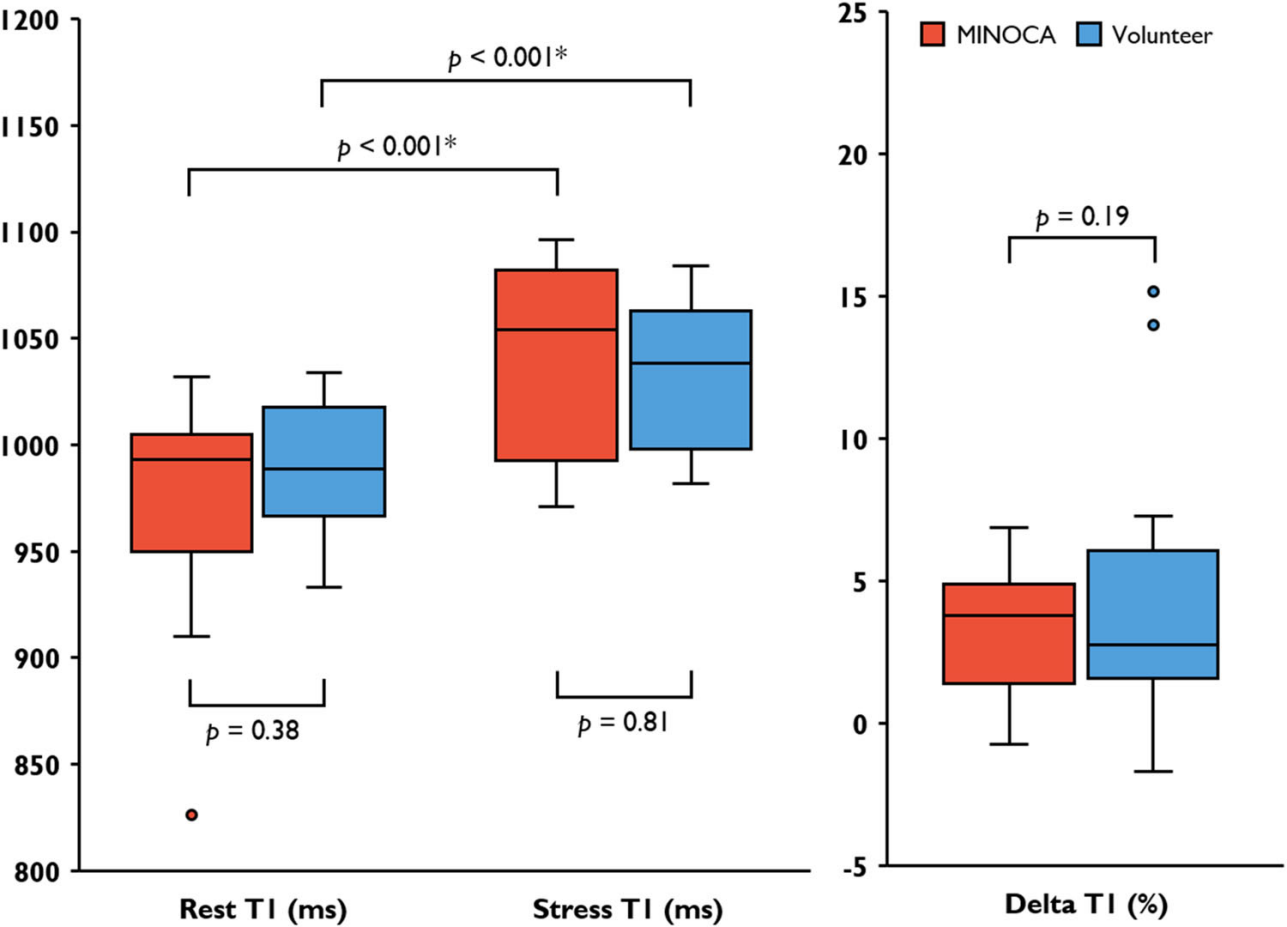
Global Transmural Native T1 in Patients with MINOCA and volunteers. Comparison of absolute native T1 in rest and stress between patients with previous MINOCA and volunteers, including delta T1. Points denote outlier values.

**Figure 3:**
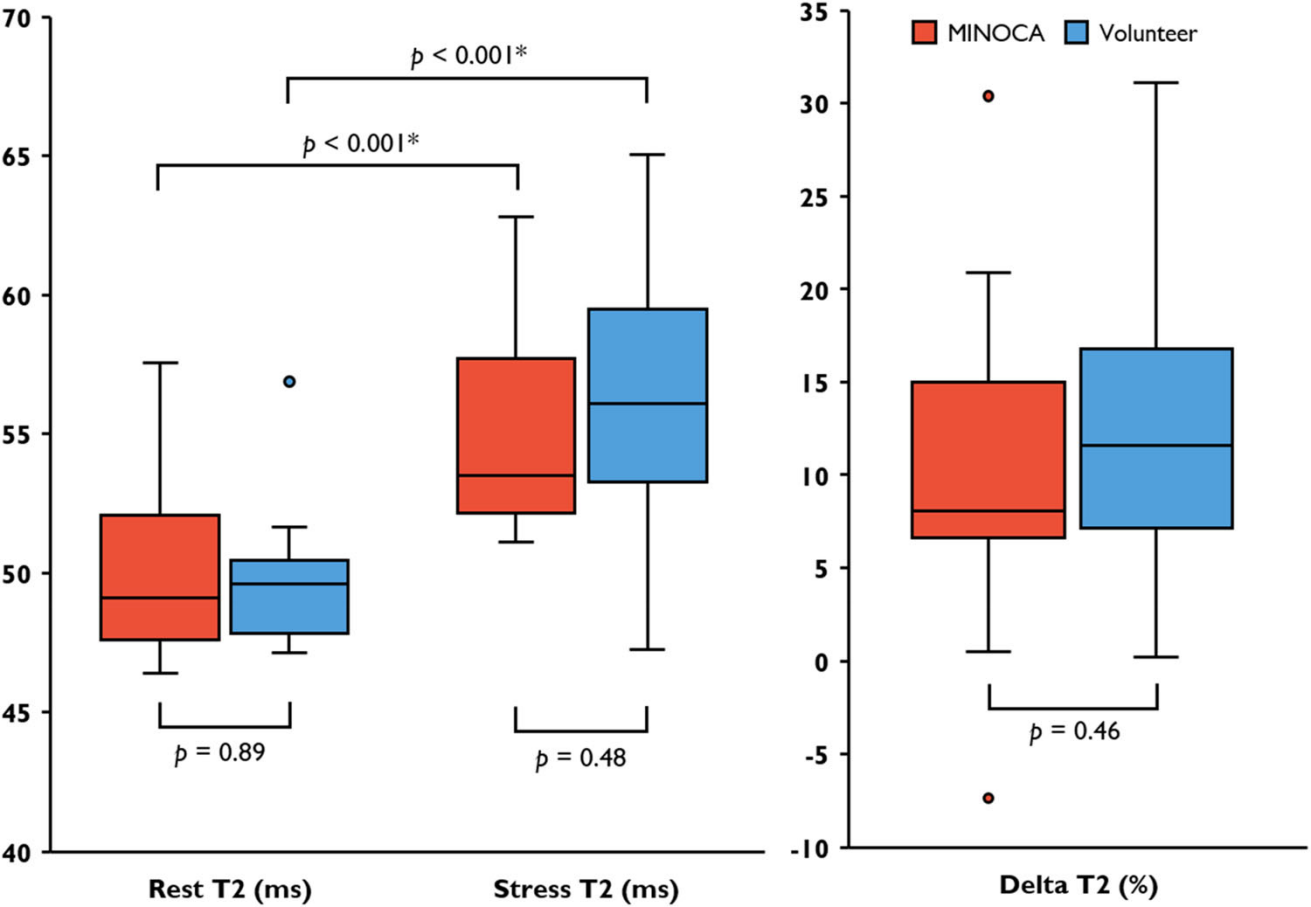
Global Transmural Native T2 in Patients with MINOCA and volunteers. Comparison of absolute native T2 in rest and stress between patients with previous MINOCA and volunteers, including delta T2. Points denote outlier values.

There were no differences in global transmural rest native T1 (975.4 ± 53.2 vs 989.3 ± 30.3 ms, *p* = 0.385), stress native T1 (1036.7 ± 44.1 vs 1033.2 ± 32.7 ms, *p* = 0.811), rest native T2 (49.9 ± 3.1 vs 49.8 ± 2.3 ms, *p* = 0.890) or stress native T2 (54.9 ± 3.8 vs 56.1 ± 4.7 ms, *p* = 0.478) between patients with MINOCA and a previously normal CMR and healthy volunteers (Figures 2 and 3) (Table 2). The relative changes in global transmural T1 and T2 from rest to stress, ΔT1 and ΔT2 respectively, did not differ between patients with MINOCA and volunteers. There were no significant differences in global subendocardial native T1 or T2 between patients and volunteers (Table 2).

**Table 2:** Findings of native and perfusion mapping.

	Transmural			Subendocardial		
	MINOCA (*n* = 15)	Volunteers (*n* = 15)	*p*	MINOCA (*n* = 15)	Volunteers (*n* = 15)	*p*
Native T1 rest, ms (global)	975.4 ± 53.2	989.3 ± 30.3	0.385	984.1 ± 51.7	992.2 ± 26.8	0.596
Native T1 rest, ms (mid‐slice)	967.0 ± 59.1	977.5 ± 24.7	0.529	972.4 ± 60.0	981.6 ± 24.6	0.585
Native T1 stress, ms	1036.7 ± 44.1	1033.2 ± 32.7	0.811	1025.7 ± 40.0	1023.6 ± 31.6	0.871
Δ T1, % (global)	6.5 ± 5.4	4.5 ± 2.2	0.193	4.4 ± 4.6	3.2 ± 2.4	0.377
Δ T1, % (mid only)	6.6 ± 5.5	5.0 ± 2.1	0.303	5.8 ± 6.0	4.3 ± 2.3	0.382
Native T2 rest, ms (global)	49.9 ± 3.1^a^	49.8 ± 2.3	0.890	49.3 ± 2.7^a^	48.8 ± 3.3	0.670
Native T2 rest, ms (mid‐slice)	49.4 ± 2.8^a^	48.6 ± 3.4	0.483	48.8 ± 3.2^a^	47.7 ± 3.5	0.390
Native T2 stress, ms	54.9 ± 3.8^a^	56.1 ± 4.7	0.478	53.2 ± 4.5^a^	53.9 ± 4.4	0.685
Δ T2, % (global)	10.3 ± 9.0^a^	12.5 ± 7.5	0.464	8.1 ± 10.4^a^	10.4 ± 7.5	0.498
Δ T2, % (mid‐slice)	10.4 ± 7.7^a^	12.1 ± 7.6	0.553	9.4 ± 11.2^a^	13.1 ± 7.4	0.302
ECV, %	26.7 ± 4.0	27.4 ± 2.3	0.562	28.1 ± 4.4	29.8 ± 4.1	0.298
Rest perfusion, mL/min/g	0.952 ± 0.198	1.288 ± 0.630	0.056	0.950 ± 0.172	1.299 ± 0.650	0.054
Stress perfusion, mL/min/g	2.86 ± 0.88	3.55 ± 0.69	**0.023**	2.64 ± 0.81	3.47 ± 0.78	**0.008**
MPR	3.07 ± 0.94	3.10 ± 1.01	0.927	2.83 ± 0.88	3.00 ± 1.01	**0.627**
Subendocardial/transmural perfusion ratio (global)				0.921 ± 0.042	0.957 ± 0.039	**0.021**

*Note*: *p*‐values denotes the independent *t*‐test, twotwo‐tailed.‐tailed. The subendocardial borders were defined as a 10% endocardial margin and a 50% epicardial margin within the delineated myocardium.

Abbreviations: ECV, extracellular volume; MPR, myocardial perfusion reserve.

a T2 maps missing for one patient due to operator dependency.

### 3.2 Myocardial perfusion

Patients with MINOCA had lower global transmural stress perfusion compared to volunteers (2.86 ± 0.88 vs 3.55 ± 0.69 ml/min/g, *p* = 0.023), as previously published (Steffen Johansson et al., 2023). These global differences in stress perfusion were more pronounced when measured in the subendocardium (2.64 ± 0.81 vs. 3.47 ± 0.78, *p* = 0.008), and the global ratio of subendocardial‐to‐transmural stress perfusion was significantly lower in patients with MINOCA compared with volunteers (0.921 ± 0.042 vs 0.957 ± 0.039, *p* = 0.021) (Table 2).

### 3.3 Late gadolinium enhancement

Ischemic late gadolinium enhancement was present in one patient who had a symptomatic MI during the initial 6 months follow‐up period. Remaining patients and volunteers had no late gadolinium enhancement.

### 3.4 Correlations between global T1‐ and T2‐mapping and perfusion

**Figure 4:**
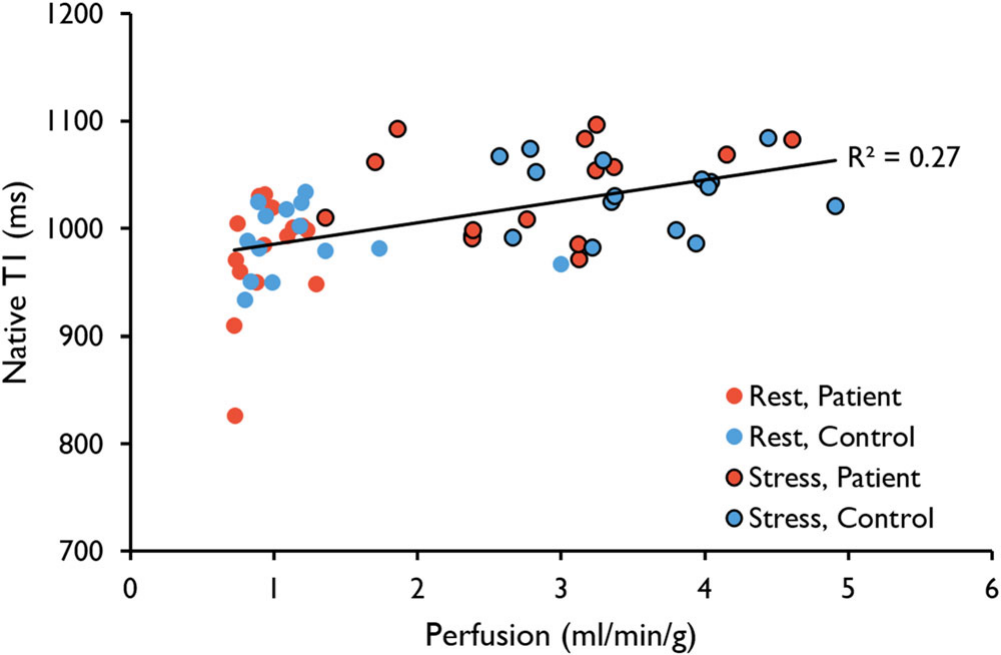
Global Correlation between Transmural Native T1 and Perfusion. Native T1 values plotted against perfusion. The trendline and R2‐value denote the correlation for all data points‐ both rest and stress in pooled patients and volunteers (R^2^ = 0.27, *p*<0.001). There was no significant correlation between rest and stress values separately.

**Figure 5:**
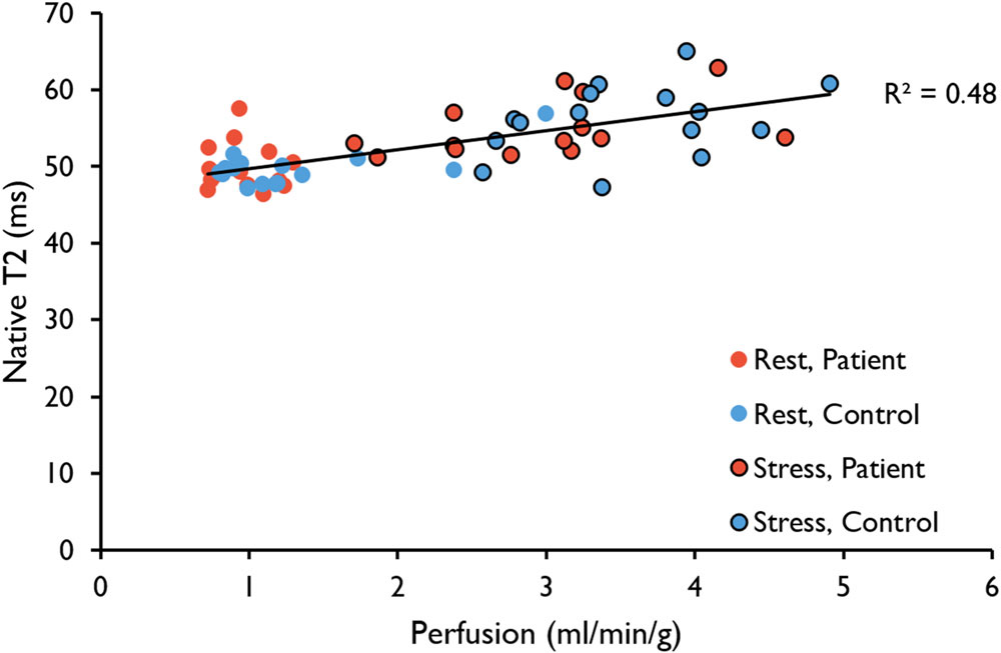
Global Correlation between Transmural Native T2 and Perfusion. Native T2 values plotted against perfusion. The trendline and R2‐value denote the correlation for all data points‐ both rest and stress in pooled patients and volunteers (R^2^ = 0.48, *p* < 0.001). There was no significant correlation between rest and stress values separately.

**Figure 6:**
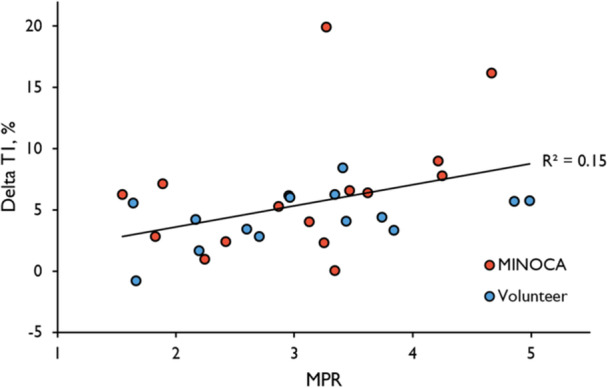
Correlation between global transmural ΔT1 and MPR. The global ΔT1 correlated positively with the MPR in pooled data from both patients with MINOCA and healthy volunteers (R^2^ = 0.15, *p* = 0.032). There were no significant correlations between global ΔT1 and MPR in patients with MINOCA or healthy volunteers alone. The correlations for each group separately are presented in Table 3.

In pooled global transmural rest‐ and stress data, native T1 correlated positively with myocardial perfusion for both the patient‐ (R^2^ = 0.33, *p* < 0.001) and volunteer (R^2^ = 0.24, *p* < 0.006) groups separately, as did the native T2 (R^2^ = 0.42, *p* < 0.001 for patients, R^2^ = 0.52, *p* < 0.001 for volunteers) (Table 3). In pooled transmural global patient and volunteer data, native T1 (R^2^ = 0.27, *p* < 0.001) (Figure 4) and native T2 (R^2^ = 0.48, *p* < 0.001) (Figure 5) both correlated positively with myocardial perfusion. The global transmural ΔT1 correlated with the MPR in pooled patient‐ and volunteer data (R^2^ = 0.15, *p* < 0.05) (Figure 6) (Table 3).

**Table 3:** Global correlations between transmural native T1‐ and T2 and myocardial perfusion.

			Rest perfusion, R^2^, *p*‐value	Stress perfusion, R^2^, *p*‐value	MPR R^2^, *p*‐value	[Rest] + [stress] perfusion, R^2^, *p*‐value
Patients (*n* = 15)	T1 (ms)	Rest	0.15, *p* = 0.16	0.01, *p* = 0.68	0.03, *p* = 0.51	
		Stress	0.00, *p* = 0.98	0.09, *p* = 0.27	0.08, *p* = 0.32	
		ΔT1 (%)	0.20, *p* = 0.10	0.01, *p* = 0.68	0.20, *p* = 0.10	
		[Rest] + [Stress]				**0.33,** ***p*** **< 0.001** **
	T2 (ms)	Rest^a^	0.01, *p* = 0.76	0.01, *p* = 0.72	0.00, *p* = 0.83	
		Stress^a^	0.16, *p* = 0.15	0.20, *p* = 0.11	0.01, *p* = 0.74	
		ΔT2^a^ (%)	0.17, *p* = 0.12	0.21, *p* = 0.08	0.02, *p* = 0.62	
		[Rest] + [Stress]^a^				**0.42,** ***p*** **< 0.001**,**, a
Volunteers (*n* = 15)	T1 (ms)	Rest	0.00, *p* = 0.86	0.00, *p* = 0.91	0.00, *p* = 0.86	
		Stress	0.10, *p* = 0.26	0.00, *p* = 0.86	0.07, *p* = 0.33	
		ΔT1 (%)	0.16, *p* = 0.15	0.00, *p* = 0.90	0.22, *p* = 0.08	
		[Rest] + [Stress]				**0.24,** ***p*** **= 0.006** **
	T2 (ms)	Rest	**0.44,** ***p*** **= 0.007** **	**0.27,** ***p*** **= 0.049** *	0.09, *p* = 0.26	
		Stress	**0.31,** ***p*** **= 0.030** *	0.12, *p* = 0.20	0.13, *p* = 0.19	
		ΔT2 (%)	0.07, *p* = 0.35	0.01, *p* = 0.74	0.06, *p* = 0.39	
		[Rest] + [Stress]				**0.54,** ***p*** **< 0.001** **
[Patients] + [volunteers]	T1 (ms)	Rest	0.02, *p* = 0.50	0.02, *p* = 0.49	0.01, *p* = 0.52	
		Stress	0.03, *p* = 0.34	0.02, *p* = 0.46	0.07, *p* = 0.15	
		ΔT1 (%)	0.08, *p* = 0.13	0.06, *p* = 0.20	**0.15,** ***p*** **= 0.032** *	
		[Rest] + [Stress]				**0.27,** ***p*** **< 0.001** **
	T2 (ms)	Rest^a^	0.12, *p* = 0.07	0.02, *p* = 0.51	0.03, *p* = 0.36	
		Stress^a^	**0.26,** ***p*** **= 0.005** **	**0.17,** ***p*** **= 0.028** *	0.03, *p* = 0.34	
		ΔT2^a^ (%)	0.04, *p* = 0.27	0.02, *p* = 0.43	0.00, *p* = 0.77	
		[Rest] + [Stress]^a^				**0.48,** ***p*** **< 0.001** **

^a^ Missing T2 data from 1 patient.

* Significant at the 0.05 level.

** Significant at the 0.01 level.

## 4 DISCUSSION

The present study found no differences in absolute native T1 or T2 values at rest or during adenosine stress between patients with previous MINOCA and healthy volunteers, adding to growing evidence indicating that native T1 and T2 are not sensitive enough at present to replace contrast agents in stress CMR (Aquaro & De Gori, 2025). Similar findings have been made in the setting of obstructive CAD (Borodzicz‐Jazdzyk et al., 2025; Shergill et al., 2026). However, the study demonstrates significant differences in subendocardial perfusion between groups as well as significant correlations between myocardial perfusion and native T1 and T2, suggesting that native T1 and T2 values in hyperemic conditions could act as potential surrogate measures for gadolinium‐enhanced stress perfusion maps in the future.

The patient group in the present study was chosen from a cohort of patients with MINOCA who had a normal standard clinical CMR scan in the SMINC‐2 multicentre study (Sörensson et al., 2021) as well as at 6 months follow‐up (Nickander et al., 2023). It was theorized that the initial inability to provide a diagnosis in these patients could be attributed to CMD (Sörensson et al., 2021), which ought to reduce stress perfusion and/or MPR (Kotecha et al., 2019; Rahman et al., 2021). Given that changes in native T1 and T2 correlate with myocardial perfusion in healthy subjects (Nickander et al., 2019), and that native T1 is reduced in patients with well‐controlled type 2 diabetes, possibly due to CMD (Levelt et al., 2016), we hypothesized that quantitative native T1 and T2 mapping could distinguish between patients with previous MINOCA and healthy volunteers. However, our study demonstrated no differences in native T1 and T2 between groups, suggesting that stress native T1 and T2 mapping, in their present form, cannot distinguish patients with previous MINOCA and an otherwise normal CMR examination from healthy age‐ and sex‐matched volunteers.

There are likely both biological and technical explanations for the inability of native T1 and T2 mapping to distinguish between the two groups. The total native T1 and T2 stress responses are small (approximately 5%–6% for T1 and 10%–12% for T2, as measured in the present cohort and previously reported in healthy volunteers (Nickander et al., 2019)). The between‐group differences in these stress responses are substantially smaller still, in the order of 1–2 percentage points, corresponding to approximately 15 ms for T1 and less than 1 ms for T2, which falls within the measurement variability of current mapping sequences.

Several technical factors may contribute to this. First, native T1 and T2 mapping sequences have limited precision relative to the small between‐group differences observed in this cohort (Messroghli et al., 2017). Second, stress mapping was performed in a single midventricular slice, reducing statistical power. Third, partial volume effects, particularly in the thin subendocardial layer, may dilute the signal of interest. Fourth, heart rate dependence of the MOLLI sequence may introduce additional variability during adenosine stress (Kellman & Hansen, 2014). Our findings are consistent with those of Radunski et al., who reported poor diagnostic performance of segmental T1‐reactivity compared with stress‐perfusion CMR in a cohort of 184 patients with known or suspected coronary artery disease (AUC = 0.60), concluding that a contrast‐free, clinically applicable T1‐reactivity approach has yet to be established (Radunski et al., 2022).

The correlations shown in our study between quantitative perfusion maps and native T1 and T2 affirm similar correlations previously shown in 41 healthy volunteers (Nickander et al., 2019) and in 37 patients with covid‐19 (Nickander et al., 2023), further extending the findings to a mixed cohort of 30 patients with previous MINOCA and healthy volunteers.

Subendocardial hypoperfusion is a pathophysiological hallmark of CMD (Rahman et al., 2021; Slivnick et al., 2022), and the present study found that the reduced stress perfusion was more pronounced when measured in the subendocardium, and that the ratio of subendocardial‐to‐transmural stress perfusion was significantly reduced in patients with MINOCA. This is supported by previous research demonstrating that subendocardial stress perfusion (Panting et al., 2002) and subendocardial MPR (Rahman et al., 2021) are more sensitive than transmural MPR for detecting CMD in patients with angina pectoris and non‐obstructive CAD. Myocardial injury in patients with MINOCA is known to have a predilection for the subendocardium (Fan et al., 2021; Zhao et al., 2022), likely explained by a greater distance from epicardial vessels (Stanton & Marwick, 2010) and transmural differences in vascular compliance (Algranati et al., 2011). When measured transmurally, only the absolute perfusion at stress differed significantly between groups (Steffen Johansson et al., 2023), which is supported by previous studies showing that transmural absolute stress perfusion is more sensitive than the MPR in discriminating CMD from three‐vessel disease (Kotecha et al., 2019), and more accurate than MPR at detecting obstructive CAD (Wang et al., 2024). Although the present study demonstrated statistically significant differences between groups, it is worth noting that the added clinical value is limited, with further development needed.

Further studies could utilize higher spatial resolution, as have previous studies using high‐resolution (voxel size 1.25 × 1.25 × 2.5 mm) LGE, which lead to changes in diagnosis in 45 of 172 patients with MINOCA and inconclusive findings after conventional CMR and a 29% lower rate of inconclusive final diagnosis (Lintingre et al., 2020). A similar study comparing standard (2.5 × 2.5 mm in‐plane) with high‐resolution (1.6 × 1.6 mm in‐plane) perfusion CMR in 111 patients with suspected CAD found that the latter could detect subendocardial ischemia in 279 versus 108 segments in the 70 patients who had CAD (Motwani et al., 2012).

In addition, future studies could investigate the effects of performing rest imaging prior to adenosine stress, as the use of rapid stress‐rest adenosine protocols have been treated with caution in recent European guidelines (Sciagrà et al., 2021) due to residual hyperaemic effects of adenosine, which could lead to underestimation of the myocardial flow reserve (Garefa et al., 2024).

## 5 LIMITATIONS

Results from the present study may be affected by the time elapsed from the initial index event to perfusion CMR, as many causes of MINOCA are transient in nature (Khan et al., 2022) and early diagnostic findings normalize at a 6‐month follow‐up in almost half of patients with suspected MINOCA (Nickander et al., 2023). While CMD is considered a stable condition (Del Buono et al., 2021), transient conditions such as microvascular artery spasm, estimated to be present in about 25% of patients with MINOCA (Mohri et al., 1998), may momentarily mimic stable CMD and later resolve. The present study did not include any invasive measurement of coronary microvascular disease. Adenosine is contraindicated in patients with asthma, which limits the generalisability of findings to the broader population. Future studies may consider using alternatives to adenosine, such as regadenosone, as adenosine has been demonstrated to mediate changes in native T1 and MPR through different receptors (Shah et al., 2021). Furthermore, the patients enrolled in the present study were asymptomatic at the time of follow‐up CMR, as assessed by the Seattle Angina Questionnaire. Symptomatic patients with more severe coronary microvascular dysfunction may have exhibited greater reductions in stress perfusion, and it is possible that native T1 and T2 differences may become detectable in such a cohort. Finally, although well characterised, a small cohort size and thorough inclusion criteria constitute a risk for selection bias. A post‐hoc power analysis for ΔT1 indicated that a sample size of 19 patients would be required to achieve a power of 0.8 with an alpha level of 0.05 and a beta level of 0.2.

## 6 CONCLUSIONS

Native T1‐ and T2‐mapping during adenosine stress, although correlated with quantitative myocardial perfusion, are not alone sufficiently sensitive methods for distinguishing patients with MINOCA and reduced stress perfusion from healthy volunteers.

## CONFLICT OF INTEREST STATEMENT

J.N. has received speaker compensation from Amicus unrelated to this work. The rest of the authors report nothing to disclose.

## Supporting information

Supporting file 1.

## Data Availability

The data that support the findings of this study are available from the corresponding author upon reasonable request.
