# Supplementary material for: Stress native mapping does not distinguish patients with previous myocardial infarction with non‐obstructive coronary arteries from healthy volunteers
Source: Clin Physiol Funct Imaging. 2026 Jun 5;46(4):e70070. doi: 10.1111/cpf.70070 (PMC13238311; doi:10.1111/cpf.70070)
Supplement: Supplementary file 1 — Supporting file 1. [file CPF-46-0-s001.docx]

**Table 4 (Supplementary). Segments excluded**

| Map, total segments | Patient segments excluded (%) | Volunteer segments excluded (%) |
| --- | --- | --- |
| Native T1 rest, 240 | 0 (0%) | 0 (0%) |
| Native T1 stress, 90 | 1 (1.1%) | 0 (0%) |
| Native T2 rest, 240 | 16 (6.7%) | 1 (0.4%) |
| Native T2 stress, 90 | 6 (6.7%) | 0 (0%) |
| ECV, 240 | 19 (7.9%) | 37 (15.4%) |
| Perfusion rest, 240 | 4 (1.7%) | 0 (0%) |
| Perfusion stress, 90 | 1 (0.4%) | 0 (0%) |

**Table 5 (Supplementary). Inter- and intra-observer reliability**

|  | **Intra-observer^1^** | ***p*** | **Inter-observer^2^** | ***p*** |
| --- | --- | --- | --- | --- |
|  | Average measures |  | Average measures |  |
| **Native T1 rest** | 0.99 (0.96-0.99) | <0.001 | 0.99 (0.97-0.99) | <0.001 |
| **Native T1 stress** | 0.99 (0.98-0.99) | <0.001 | 0.92 (0.68-0.97) | <0.001 |
| **Native T2 rest** | 0.96 (0.48-0.99) | <0.001 | 0.95 (0.82-0.98) | <0.001 |
| **Native T2 stress** | 0.97 (0.87-0.99) | <0.001 | 0.92 (0.71-0.97) | <0.001 |
| **ECV** | 0.98 (0.94-0.99) | <0.001 | 0.92 (0.83-0.96) | <0.001 |
| **Perfusion rest** | 0.99 (0.99-1.00) | <0.001 | 0.99 (0.99-0.99) | <0.001 |
| **Perfusion stress** | 0.99 (0.99-1.00) | <0.001 | 0.99 (0.97-0.99) | <0.001 |
| **MPR** | 0.99 (0.99-1.00) | <0.001 | 0.99 (0.99-0.99) | <0.001 |

Inter- and intra-observer reproducibility calculated with the intra-class correlation coefficient (95% CI). Abbreviations: ECV: Extracellular volume. MPR: Myocardial perfusion reserve. ^1^Based on 30 patients, ^2^Based on 10 patients

Figure 7. (Supplementary) Example Delineation Images

E

B

A


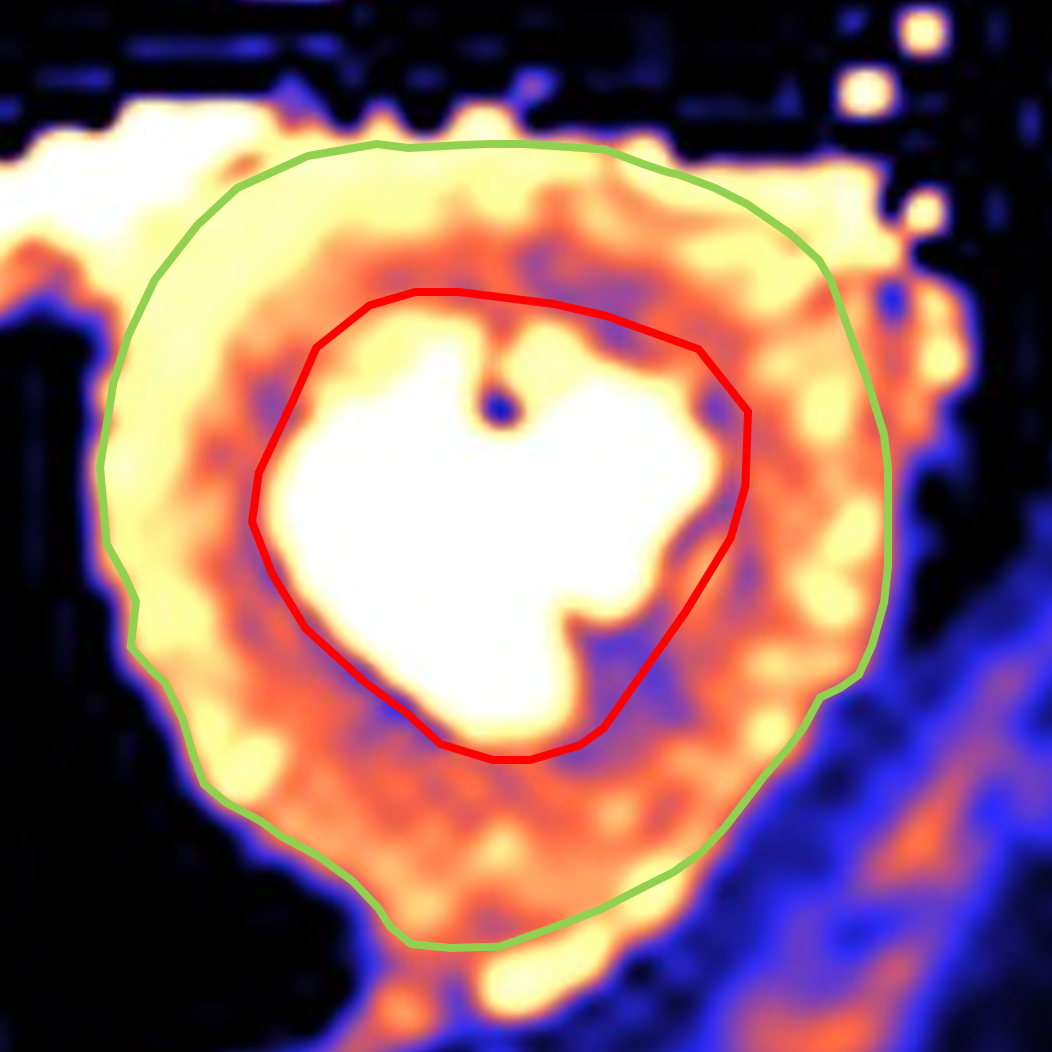

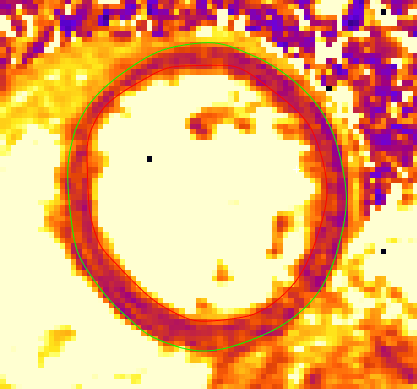

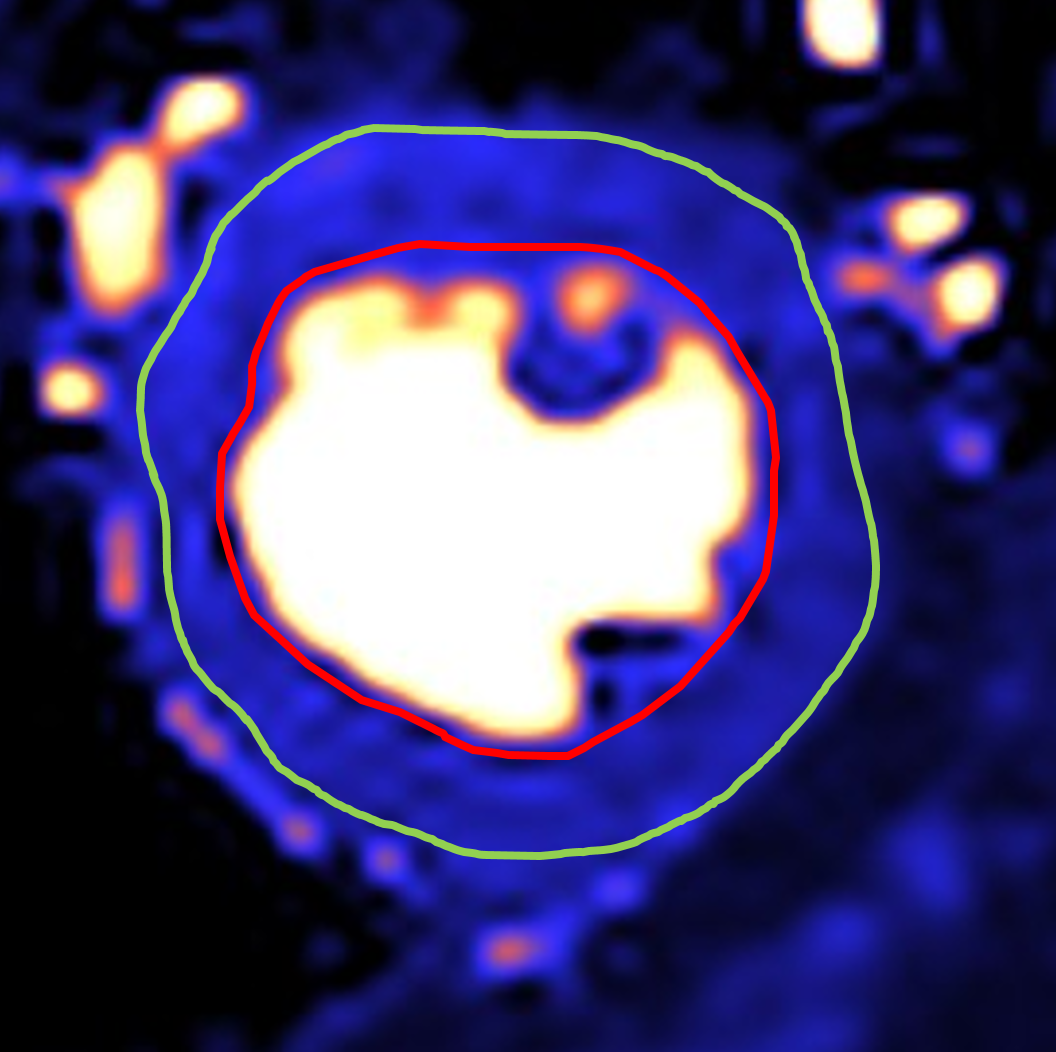

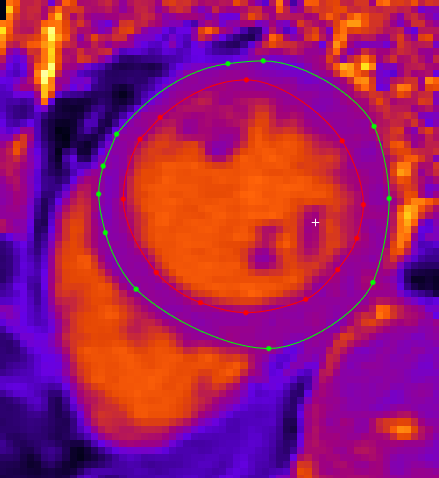

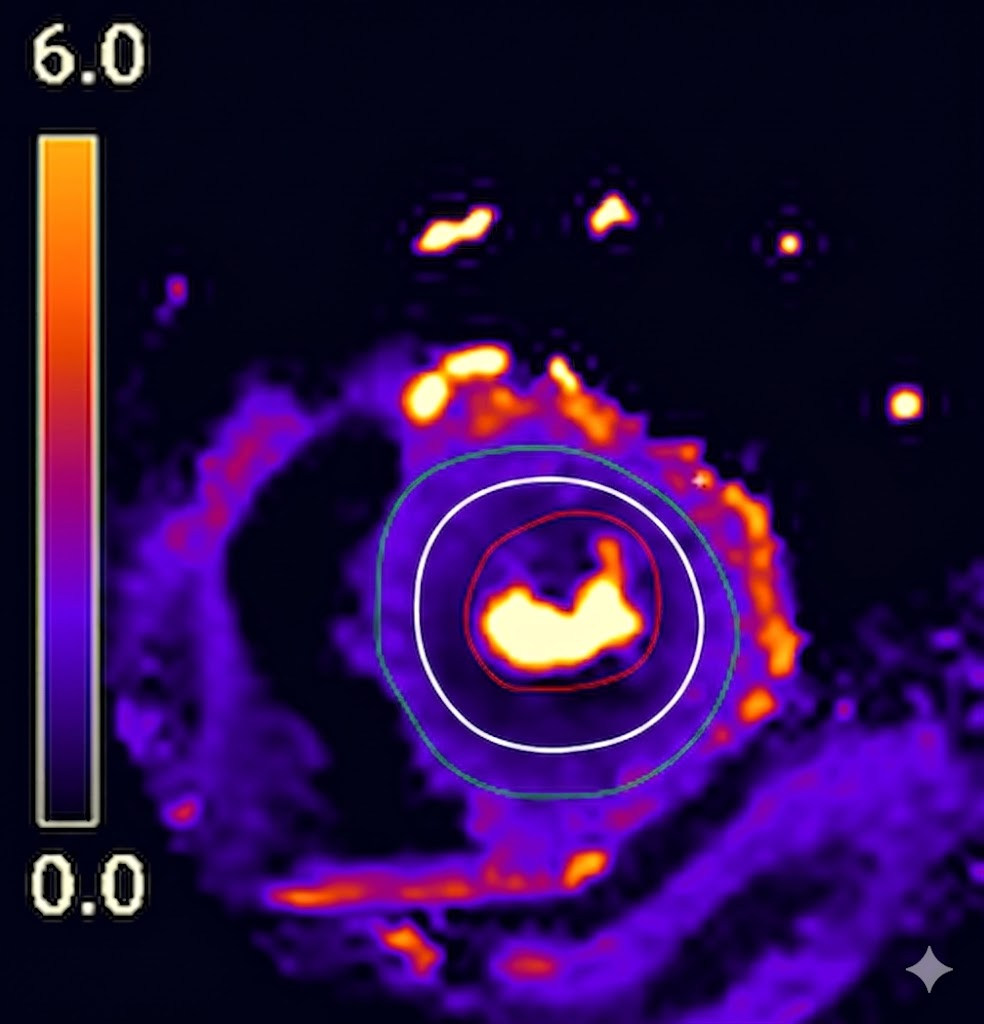


D

C

Representative example figure of delineations in perfusion, T1 and T2 slices, with endocardial borders shown in red and epicardial borders in green. The rightmost figure shows 10% endo- and epicardial erosion margins as yellow lines. The top left images demonstrate example stress mapping images with signs of hypoperfusion.
